# N^6^-Methyladenosine and Viral Infection

**DOI:** 10.3389/fmicb.2019.00417

**Published:** 2019-03-05

**Authors:** Wei Dang, Yan Xie, Pengfei Cao, Shuyu Xin, Jia Wang, Shen Li, Yanling Li, Jianhong Lu

**Affiliations:** ^1^Department of Hematology, Xiangya Hospital, Central South University, Changsha, China; ^2^Department of Microbiology, Cancer Research Institute, School of Basic Medical Science, Central South University, Changsha, China

**Keywords:** m^6^A, virus, infection, immune, viral life cycle

## Abstract

N^6^-methyladenosine (m^6^A), as a dynamic posttranscriptional RNA modification, recently gave rise to the field of viral epitranscriptomics. The interaction between virus and host is affected by m^6^A. Multiple m^6^A-modified viral RNAs have been observed. The epitranscriptome of m^6^A in host cells are altered after viral infection. The expression of viral genes, the replication of virus and the generation of progeny virions are influenced by m^6^A modifications in viral RNAs during virus infection. Meanwhile, the decorations of m^6^A in host mRNAs can make viral infections more likely to happen or can enhance the resistance of host to virus infection. However, the mechanism of m^6^A regulation in viral infection and host immune response has not been thoroughly elucidated to date. With the development of sequencing-based biotechnologies, transcriptome-wide mapping of m^6^A in viruses has been achieved, laying the foundation for expanding its functions and corresponding mechanisms. In this report, we summarize the positive and negative effects of m^6^A in distinct viral infection. Given the increasingly important roles of m^6^A in diverse viruses, m^6^A represents a novel potential target for antiviral therapy.

## Introduction

Among the diverse layers of epigenetic regulation, modifications of DNA and proteins have been explored in depth; however, RNA modification is a relatively new field (He, 2010). More than 100 posttranscriptional covalent modifications have been identified on RNA transcripts from various organisms, including viruses, yeast, and mammals (Meyer et al., 2012; Mamadou et al., 2013; Tan and Gao, 2018). The most prevalent modification of internal messenger RNA in eukaryotes and in nuclear-replicating viruses is the addition of a methyl group to the N^6^ position of adenosine, known as m^6^A (Dominissini et al., 2012; Meyer and Jaffrey, 2014). In 1975, m^6^A was first reported to exist in cellular mRNAs, and it was reported that there are three internal m^6^A residues on the average ∼2.2 kb cellular transcript (Desrosiers et al., 1975; Kennedy et al., 2017). Furthermore, highly decorated and regulated transcripts might include ten or more m^6^A groups (Linder et al., 2015). Similar to DNA methylation, the m^6^A modification is reversibly catalyzed by corresponding enzymes (Li et al., 2018). Despite the discovery of m^6^A decades ago (Desrosiers et al., 1974; Adams and Cory, 1975; Wei et al., 1975), the related signaling pathways it is involved in and the biological roles it plays were not fully described until recently (Nilsen, 2014; Cao et al., 2016). Meanwhile, with the development of efficient methods of m^6^A detection and subsequent analysis, increasing biological functions are being elucidated (Meyer et al., 2012; Li et al., 2015). m^6^A has been reported to control the fate of modified RNAs at multiple steps, including RNA splicing (Dominissini et al., 2012), mRNA stability (Li and Mason, 2014), cap-independent translation (Meyer et al., 2015), and miRNA biogenesis (Alarcon et al., 2015b). m^6^A-decorated RNAs participate in many biological processes, such as stress responses (Zhou et al., 2015), cellular reprogramming (Chen T. et al., 2015), circadian cycle (Fustin et al., 2013), stem cell differentiation (Yue et al., 2015), fertility (Zhao and He, 2017), and cancer (Pan et al., 2018).

Viruses, as a type of organism, were found to be modified by m^6^A in their genomic RNAs (Zhou et al., 2010; Courtney et al., 2017). Earlier studies have shown that some viruses, such as simian virus 40 (Lavi and Shatkin, 1975), influenza A virus (Krug et al., 1976), adenovirus (Sommer et al., 1976), avian sarcoma virus (Dimock and Stoltzfus, 1977), and RSV (Kane and Beemon, 1985) have m^6^A residues in their mRNAs. Narayan and colleagues performed the biochemical analysis of different influenza virus mRNAs, to detect the distribution of m^6^A and showed that the amount of m^6^A in different hemagglutinin (HA) mRNAs varied (Narayan et al., 1987). Those authors attempted to explore the functions of m^6^A in RNA splicing and translation, but the experimental conditions and relevant knowledge of m^6^A at that time were not enough. The specific sites of m^6^A were not mapped, and the functions of m^6^A in IAV remained vague for decades since the restriction with techniques and knowledge of the methyltransferases, demethylases, and m^6^A reader proteins then. Transcriptome-wide mapping of m^6^A was available after N^6^-methyladenosine-sequencing (m^6^A-seq) was developed by two independent research teams in Dominissini et al. (2012) and Meyer et al. (2012). In the following years, m^6^A has attracted the attention of scientists to elucidate the role it plays in viral epitranscriptomics (Gokhale and Horner, 2017; Kennedy et al., 2017). Immediately following m^6^A-seq, new technologies to unravel the m^6^A epitransctiptome were developed, including PA-m^6^A-seq, miCLIP, m^6^A-LAIC-seq, microarray, and SELECT (Chen K. et al., 2015; Li et al., 2015; Linder et al., 2015; Molinie et al., 2016; Xiao et al., 2018). The advantages and disadvantages of these techniques were well summarized in a review by Tan and Gao (2018). Based on these sequencing data, some bioinformatics tools were developed to help researchers investigate the potential functions and mechanisms of m^6^A modification. At present, most of the m^6^A decoration profiles have been available in some databases. A brief description of these databases related to m^6^A research are summarized in Table 1. Moreover, some computational m^6^A site predictors have been constructed, such as SRAMP (Zhou et al., 2016), m6Apred (Chen W. et al., 2015) and RNA-MethylPred (Jia et al., 2016). Although few studies have been done using these bioinformatics tools on the role of m^6^A in viral infection currently, they would be helpful for m^6^A prediction prior to experimental study. With the help of all these advanced technologies, a number virus types were subjected to transcriptome-wide mapping of m^6^A. Gokhale et al. (2016) mapped the m^6^A sites within the viral RNA genomes of the flaviviridae family of viruses, including HCV, ZIKV, DENV, YFV, and WNV. These researchers verified that m^6^A negatively regulated HCV infection by using m^6^A-abrogating mutations in HCV E1 (Gokhale et al., 2016). In addition, other viruses, such as influenza A virus (IAV) (Courtney et al., 2017), Kaposi’s Sarcoma-Associated Herpesvirus (KSHV) (Ye et al., 2017), Human Immunodeficiency Virus-1 (HIV-1) (Tirumuru et al., 2016), Simian Virus 40 (SV40) (Tsai et al., 2018), Hepatitis B Virus (HBV) (Imam et al., 2018), and Enterovirus 71 (EV71) (Hao et al., 2019) have been subjected to transcriptome-wide mapping of m^6^A and reported for their different roles in the life cycles of the viruses.

**Table 1:** Brief introduction of bioinformatics database on m^6^A research.

Database name	Description	Data sources	URL	Reference
RNAMethPre	A user-friendly web server for m^6^A site prediction and query for human, mouse, and mammal, broadly.	Single-base resolution m^6^A site data generated using the miCLIP approach.	http://bioinfo.tsinghua.edu.cn/RNAMethPre/index.html	Xiang et al., 2016
m^6^AVar	It is a comprehensive database of m^6^A-associated variants that potentially influence m^6^A modification, which will help to interpret variants by m^6^A function.	miCLIP/PA-m^6^A-seq experiments, MeRIP-Seq experiments and transcriptome-wide predictions.	http://m6avar.renlab.org/	Zheng et al., 2018
RMBase v2.0	It is a comprehensive database that integrates epitranscriptome sequencing data for the exploration of post-transcriptional modifications of RNAs and their relationships with miRNA binding events, disease-related single nucleotide polymorphisms (SNPs) and RNA-binding proteins (RBPs).	High-throughput epitranscriptome sequencing data that covered 13 species including humans, mice, zebrafish, yeast, etc.	http://rna.sysu.edu.cn/rmbase/	Xuan et al., 2018
MeT-DB v2.0	MeT-DB V2.0 is a comprehensive and significantly enhanced database collecting and integrating more MeRIP-seq samples; It focus more on helping elucidate context-specific m^6^A functions.	185 MeRIP-seq samples which come from 26 independent studies covering 7 species.	https://whistle-epitranscriptome.com/metdb_v2/html/genome_browser.php	Liu et al., 2018

Our review summarizes the recent advances in related literature in this relatively new emerging field and discusses the different functions and relevant mechanisms of m^6^A in the biological processes of different types of viral infections.

## m^6^A and Its Related Molecular Mechanisms

The associated mechanisms that regulate m^6^A involve proteins working as writers, erasers, readers, and anti-readers (Edupuganti et al., 2017; Li et al., 2018). M^6^A decoration is not randomly distributed in RNA transcripts (Batista, 2017), which was found to occur on the consensus RNA motif of RRACH (R = A or G;H = A, U, or C), and preferentially center on specific transcript landmarks such as near 3′ untranslated regions (3′ UTRs) and stop codons or in long exons (Yang et al., 2018). m^6^A is added cotranscriptionally to nuclear pre-mRNAs by a multicomponent protein complex consisting of catalytic subunit Methyltransferase Like 3 (METTL3) (Yao et al., 2018), RNA-binding platform METTL14 (Kobayashi et al., 2018), cofactors Wilms tumor 1-associated protein (WTAP) and KIAA1429 (Schwartz et al., 2014; Scholler et al., 2018), two novel subunits of the methyltransferase complex RBM15 (Patil et al., 2016), and zinc finger CCCH domain-containing protein 13 (ZC3H13) (Guo et al., 2018; Knuckles et al., 2018; Wen et al., 2018). As the core methyltransferase subunit, METTL3 is a strongly conserved protein (Bujnicki et al., 2002; Yang et al., 2018) and has been demonstrated to selectively methylate the GAC or AAC motifs in synthetic single-stranded RNA *in vitro* (Rottman et al., 1994; Kan et al., 2017). METTL14 is also highly conserved in mammals and can form a stable protein heterodimer with METTL3 (Liu et al., 2014; Ping et al., 2014; Wang Y. et al., 2014). Wang et al. studied the crystal structure of Mettl3-Mettl14 complex and formulated that Mettl14 has a degenerate active site and is unavailable for catalysis (Wang et al., 2016). Mettl14 has a structural role that could offer an RNA-binding scaffold, allosterically activating and enhancing Mettl3’s catalytic function (Sledz and Jinek, 2016). As a regulatory subunit of the m^6^A methyltransferase complex, WTAP allows METTL3/METTL14 to interact with messenger RNAs in the nucleus to improve m^6^A modification efficiency (Liu et al., 2014; Ping et al., 2014). WTAP is required for the localization of the METTL3-METTL14 complex into nuclear speckles that are enriched with various precursor messenger RNA (pre-mRNA) processing factors (Ping et al., 2014). The depletion of KIAA1429 in human A549 cells results in a fourfold reduction of m^6^A abundance (Schwartz et al., 2014), suggesting a significant regulatory effect in the writer complex. Recently, RBM15 and its paralog RBM15B were shown to be members of the m^6^A methyltransferase complex that recruit the METTL3/14 protein complex to specific sites in RNA for the selective methylation (Patil et al., 2016). More recently, three research teams demonstrated that ZC3H13 was another member of the m^6^A writer complex and modulated m^6^A methylation (Guo et al., 2018; Knuckles et al., 2018; Wen et al., 2018). METTL16 is cognate with METTL3, was reported to control the cellular SAM level and catalyze the m^6^A group onto the U6 small nuclear RNA (Pendleton et al., 2017). More subunits of the m^6^A methyltransferase complex might be explored to achieve accurate posttranscriptional RNA regulation through selectively recognizing candidate m^6^A sites.

The removal of m^6^A from the decorated mRNA is catalyzed by the demethylase FTO or a-ketoglutarate-dependent dioxygenase AlkB homolog 5 (ALKBH5) (Jia et al., 2011; Zheng et al., 2013). Jia et al. (2011) found that FTO could efficiently demethylate m^6^A in RNA *in vitro*. They used siRNA mediated knockdown of FTO and then detected an increased level of m^6^A in mRNA, whereas the overexpression of FTO resulted in a decreased level of m^6^A in human HeLa cells. In 2013, another m^6^A demethylase, ALKBH5 was identified and it exhibited m^6^A demethylation efficiency comparable to that of FTO (Zheng et al., 2013). A recent report showed that ALKBH5-mediated m^6^A elimination in the nucleus of spermatocytes and round spermatids was elementary for correct splicing and the production of longer 3′-UTR mRNAs (Tang et al., 2018).

The function of m^6^A modification on target mRNAs is thought to be mediated by “reader” proteins (Riquelme-Barrios et al., 2018). To date, several m^6^A reader proteins have been identified in mammalian cellular extracts using affinity chromatography combined with mass spectrometry (Dominissini et al., 2012). Among the “reader” proteins, the YT521-B homology (YTH) family of proteins have been very well studied (Meyer and Jaffrey, 2017; Patil et al., 2018). There are three YTHDF (YTH domain family) members localized in the cytoplasm, including YTHDF1, YTHDF2, and YTHDF3 (Dominissini et al., 2012; Wang X. et al., 2014; Wang X. et al., 2015; Shi et al., 2017), and two YTHDC (YTH domain containing) proteins, YTHDC1 located in the nucleus (Xu et al., 2014) and YTHDC2 located in the cytoplasm (Morohashi et al., 2011). YTHDF proteins contain a conserved YTH RNA-binding domain that is inclined to bind the m^6^A-targeted RNAs and a N/P/Q-rich region that is correlated with different RNA-protein complexes (Fu et al., 2014). YTHDF1 has been demonstrated to interact with the translation initiation machinery and enhance the translational efficiency of its mRNA targets (Wang X. et al., 2015). The function of YTHDF2 was the induction of mRNA degradation (Wang X. et al., 2014). It has been shown that YTHDF2 accelerates the degradation of m^6^A-containing RNAs by directly recruiting the CCR4-NOT deadenylase complex (Du et al., 2016). YTHDF3 has been shown to promote the function of both YTHDF1 and YTHDF2. When it cooperates with YTHDF1, YTHDF3 can favor mRNA translation by the interactions with some ribosomal proteins (Li et al., 2017; Shi et al., 2017). Moreover, when it is associated with YTHDF2, YTHDF3 could participate in mRNA decay through direct relations with YTHDF2 (Shi et al., 2017). The nuclear m^6^A reader YTHDC1 was demonstrated to promote exon inclusion by assisting the splicing factor SRSF3 during its recruitment, while blocking the binding of SRSF10 (Kasowitz et al., 2018). Another study in HeLa cells showed that the association of YTHDC1 with SRSF3 and NXF1 could promote the nuclear export of m^6^A-target mRNAs (Roundtree et al., 2017b). YTHDC2, was identified to favor translational efficiency while decreasing the abundance of m^6^A-containing mRNAs (Hsu et al., 2017). YTHDC2 is a relatively large protein molecule (∼160 kDa) and contains many helicase domains and two ankyrin repeats. These special structural features might allow YTHDC2 to possess multiple functions, including regulating effects on RNA binding and RNA structure, and binding with or the recruitment of other interacting proteins (Tanabe et al., 2016; Hsu et al., 2017). A study reported that YTHDC2 lacked the m^6^A binding activity in HEK cells (Patil et al., 2016, 2018). This finding suggests that YTHDC2 might have indirect effects in regulating m^6^A-modified RNAs through interaction with other factors. In addition to the YTH family members, there are other proteins that have been identified to recognize and bind to m^6^A. The eukaryotic initiation factor 3 (eIF3) complex, interacts with m^6^A-containing 5′UTR through a multisubunit interface to directly recruit the 40S preinitiation complex to the 5′UTR of target-mRNAs to stimulate translation initiation (Meyer et al., 2015). hnRNPA2/B1 was shown to bind m^6^A-modified RNAs to regulate splicing and microRNA maturation (Alarcon et al., 2015a). Fragile X Mental Retardation Protein (FMRP) was also demonstrated to bind m^6^A-containing transcripts in a sequence context-dependent manner (Edupuganti et al., 2017). Recently, insulin growth factor 2 binding proteins, IGF2BP 1, 2, and 3, were shown to promote stability and storage of mRNAs through binding target m^6^A mRNAs under normal and stress conditions (Huang et al., 2018).

**Figure 1:**
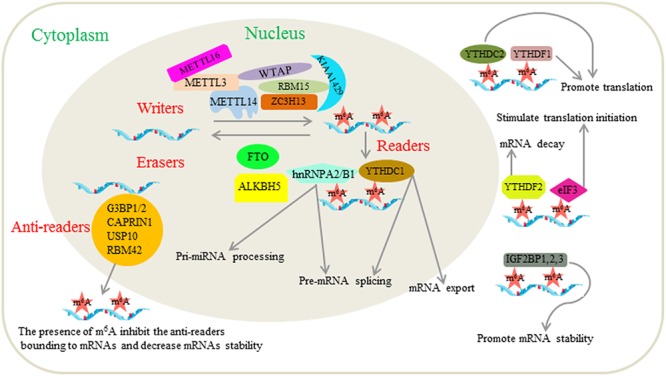
Related mechanisms and functions of m^6^A modification in mRNAs. The m^6^A modification is regulated by the “writers,” “erasers,” “readers” and “anti-readers.” Writers are composed of METTL3, METTL14, WTAP, KIAA1429, ZC3H13, RBM15, and METTL16, which have been reported to induce m^6^A RNA methylation. Erasers are m^6^A demethylases including FTO and ALKBH5. Readers are proteins that bind to m^6^A modified mRNAs and play corresponding roles. Those proteins that have been identified as readers to date include YTHDF1, YTHDF2, YTHDF3, YTHDC1, YTHDC2, eIF3, IGF2BP1, IGF2BP2, IGF2BP3, FMRP, and hnRNPA2/B1. The functions of m^6^A are related to almost all stages in deciding the fate of mRNAs including pre-mRNA splicing, pri-miRNA processing, mRNA export, mRNA stability, translation modulation and mRNA degradation. Anti-readers are proteins that preferentially bind to mRNAs in the absence of m^6^A, such as G3BP1/2, CAPRIN1, USP10, and RBM42.

Furthermore, researchers have found a new m^6^A-regulated protein, referred to as m^6^A-repelled proteins or anti-readers, during the study of proteomics that interact with m^6^A. These researchers found that m^6^A disrupts RNA binding by the stress granule proteins G3BP1/2, CAPRIN1, USP10 and RBM42 (Arguello et al., 2017). A study has confirmed that the presence of m^6^A in mRNA could decrease mRNA stability by interfering with the binding of G3BP1 and G3BP2 (Edupuganti et al., 2017). This phenomenon reveals an additional function of m^6^A in RNA metabolism. We provide a picture (Figure 1) as an overview of the associated machinery and molecular functions of m^6^A.

## Role of m^6^A in the Infection of RNA Viruses

The specific functions and mechanisms of m^6^A in RNA viruses are not well-known, although m^6^A has long been known to be present in some RNA viruses, such as IAV (Krug et al., 1976), RSV (Kane and Beemon, 1985), B77 avian sarcoma virus (Stoltzfus and Dimock, 1976; Dimock and Stoltzfus, 1977) and feline leukemia virus (Thomason et al., 1976). In recent years, technological advances have made m^6^A a focus of research in elucidating the role of this RNA modification in viral epitranscriptomics. Herein we summarize and describe the role of m^6^A in RNA viruses including IAV, HIV-1, HCV, ZIKV, VSV, and EV71.

As the first virus found to express mRNAs bearing internal m^6^A groups, Influenza A virus (IAV) was mapped for the sites of m^6^A by Courtney et al. in both the IAV mRNA and vRNA strands, and it was demonstrated that m^6^A modification increases viral RNA expression in *cis* (Courtney et al., 2017). Those authors used two methods to inhibit m^6^A modification in A549 cells infected by IAV. One method used a non-toxic dose of DAA treatment (an inhibitor of m^6^A addition), and the other used knockout of METTL3 through gene editing with CRISPR/Cas. The results showed that both methods could reduce the expression of the IAV proteins NS1 and M2. However, when the m^6^A reader YTHDF2 was ectopically overexpressed, increased IAV replication and infectious particle production were found. Moreover, they used synonymous mutations to remove m^6^A on both strands of the hemagglutinin (HA) segment, and found that IAV HA m^6^A mutants revealed reduced pathogenicity in mice. The findings confirmed that the addition of m^6^A residues in IAV transcripts could enhance viral gene expression.

Prior to this review, four reports have studied the involvement of m^6^A in HIV-1 infection. Three excellent reviews comprehensively compared and summarized the functions and mechanisms of m^6^A modification in the HIV-1 life cycle (Gonzales-van Horn and Sarnow, 2017; Riquelme-Barrios et al., 2018; Tan and Gao, 2018). Despite some agreed-upon conclusions, there are unconformities in the locations, effects, and mechanisms of m^6^A in HIV-1 RNA in these studies. Herein, a table was created to allow readers to understand functions of m^6^A more clearly and intuitively in the four different articles (Table 2).

**Table 2:** Comparison of m^6^A functions in HIV-1 life cycle.

Key conclusions	Mechanisms of action	Main m^6^A sites	Cell types used	m^6^A detection technologies	Reference
m^6^A modification, and the resultant recruitment of YTHDF proteins, are major positive regulators of HIV-1 mRNA expression.	The m^6^A abundant sites recruit the cellular YTHDF m^6^A “reader” proteins to enhance HIV-1 protein and RNA expression, and virus replication	HIV-1 3’UTR	Human CD4+ CEMSS T-cells infected with HIV-1 NL4.3 genome, HIV-1-expressig 293T cells	PA-m^6^A-seq; PAR-CLIP	Kennedy et al., 2016
m^6^A modification of HIV-1 RNA increase HIV-1 Gag protein expression; YTHDF proteins inhibited HIV-1 post-entry infection	YTHDF1–3 proteins inhibit HIV-1 infection by blocking viral reverse transcription and promoting degradation of viral RNA	5’UTR, 3’UTR and several internal positions of HIV-1	HIV-1-NL4.3 infected Jurkat cells, primary CD4+ T-cells, HEK293T cells and HeLa cells	m^6^A-seq; CLIP-seq; LC-MS	Tirumuru et al., 2016
The addition of m^6^A group in HIV-1 gRNAs enhance HIV-1 infection and viral replication	The presence of m^6^A favor the binding of Rev to the RRE in HIV-1 infected cells	In coding and non-coding regions, splicing junctions, and splicing regulatory sequences of HIV-1	MT4 T-cells infected with HIV-1 LAI strain, HEK293T cells	MeRIP-seq	Lichinchi et al., 2016a
YTHDF1-3 proteins inhibit HIV-1 infection and viral production	m^6^A reader proteins YTHDF1–3 inhibit HIV-1 infection by decreasing viral gRNA and early reverse transcription products	Undetected	HeLa or CD4+ cells overexpressing each YTHDF protein infected with HIV-1 NL4.3, HEK293T cells	None	Lu et al., 2018

RNA-based regulation of HCV plays an essential role in its infection (Wang X. et al., 2012), for example the liver-specific microRNA 122 has been proved to facilitate replication of the viral RNA (Jopling et al., 2005; Cuypers et al., 2016). Recently, Gokhale et al. carried out m^6^A analyses in cells infected with HCV and demonstrated that the HCV RNA genome was modified by m^6^A (Gokhale et al., 2016). The abundance of HCV NS5A protein was significantly increased through siRNA mediated simultaneous depletion of m^6^A methyltransferases METTL3 and METTL14 in Huh7 cells infected with HCV. In contrast, HCV NS5A levels were decreased when the m^6^A demethylase FTO was depleted. Considering that CD81 is an essential entry factor but has no effects on other steps of HCV’S life cycle (Zhang et al., 2004), Huh7.5 CD81 knockout (KO) cells were used to test whether the m^6^A machinery affects viral RNA replication or viral particle production. The results indicated that the m^6^A machinery modulated HCV particle production but did not regulate HCV translation or RNA replication. Furthermore, the authors found that YTHDF proteins relocalized at viral assembly sites to retain HCV RNA with m^6^A, leading to reduced HCV particle production. To further confirm this conclusion, mutations in the E1 gene of HCV to inactivate m^6^A modification were constructed marked as HCV-E1^mut^. Like the above findings, these mutations increased viral particle assembly. In brief, this article revealed that m^6^A, as a conserved regulatory mark across the HCV genome, negatively regulates HCV infection.

Like HCV, ZIKV is also a member of the Flaviviridae family. To date, there has been only one research article that reports the relevance of ZIKV to m^6^A modification. Twelve discrete m^6^A peaks were identified spanning the full length of ZIKV RNA, among which half were present in the region encoding the NS5 protein and the 3′ UTR region (Lichinchi et al., 2016b). Like the role of m^6^A in regulating the HCV life cycle, the knockdown of host methyltransferases METTL3 and METTL14 increased ZIKV production while the silencing of demethylases ALKBH5 and FTO suppressed ZIKV production. The difference was that the m^6^A machinery decreased HCV production by affecting the assembly of virus, yet m^6^A modification inhibited ZIKV production by impacting replication of ZIKV. Furthermore, YTHDF family proteins bound to ZIKV RNA and decreased the viral titer. Remarkably, YTHDF2 showed the strongest effect compared with YTHDF1 and YTHDF3. In total, these results identified that the m^6^A methylome was a new mechanism by which ZIKV interacted with the host cells and inhibited viral infection.

Because of its relatively simple structure and high replication capacity, VSV has been widely used in the study of innate immunity. After VSV infection, pattern-recognition receptors trigger activation of the type I interferon signaling pathway (Schlee and Hartmann, 2016). Since m^6^A is involved in regulating the life cycles of many viruses, whether m^6^A modification participates in the regulation of VSV infection is a question. The Cao group demonstrated in 2017 that knockdown of the m^6^A eraser ALKBH5 noticeably increased the production of type I interferons triggered by VSV infection (Zheng et al., 2017). This finding suggested that m^6^A modifications might play a negative role in the life cycle of VSV. To investigate the detailed mechanisms related to m^6^A in regulating the antiviral effects, the authors performed numerous biological experiments. They found that nuclear DDX member DDX46 recruited ALKBH5 via DDX46’s DEAD helicase domain to erase the m^6^A modification of three important antiviral transcripts *MAVS, TRAF3, and TRAF6* and therefore caused their retention in the nucleus and reduced their translation into protein; this result further caused decreased production of type I interferons. In effect, elimination of the m^6^A modification suppressed the exportation of mRNA from the nucleus and induced retention of the mRNA encoding proteins in the nucleus such as those involved in circadian rhythm (Fustin et al., 2013; Zheng et al., 2013); this phenomenon was well verified in this article. This work might promote research progress in m^6^A-modification-based regulation of gene expression in innate immunity.

As a single-stranded RNA virus which belongs to the genus Enterovirus within the family Picornaviridae, Enterovirus 71 (EV71) is one of the main pathogens that causes hand-foot-and-mouth disease (HFMD) (Wang and Li, 2018). Lately, a study reported that EV71 RNA contained m^6^A residues and the expression and localization of the m^6^A methyltransferase and demethylase were altered during viral infection (Hao et al., 2019). The genomic copy numbers of EV71 RNA were significantly decreased by silencing METTL3 gene and increased by FTO gene depletion. When two m^6^A sites were mutated in the EV71 RNA, the virus titer was significantly decreased. Besides, the authors found that METTL3 interacted with viral RdRp 3D protein and overexpression of METTL3 induced enhanced sumoylation and ubiquitination of 3D. As it has been reported that sumoylation and ubiquitination levels can enhance self-stability of 3D and facilitate EV71 replication (Liu et al., 2016), this suggested us that the replication of EV71 were also influenced through METTL3-mediated sumoylation and ubiquitination of viral 3D protein. Collectively, these results suggest that m^6^A modifications in EV71 RNA played a positive role in viral replication.

## Role of m^6^A in the Infection of DNA Viruses

DNA viruses use the host machinery to replicate in the nucleus, are likely to usurp m^6^A machinery to regulate their lifecycle, and have been reported to have m^6^A modifications in viral mRNAs such as Kaposi sarcoma–associated herpesvirus (KSHV), Simian virus 40 (SV40) and Hepatitis B virus (HBV) (Ye et al., 2017; Hesser et al., 2018; Imam et al., 2018; Tsai et al., 2018).

Kaposi’s sarcoma-associated herpesvirus (KSHV) is a carcinogenic virus associated with a variety of malignant tumors including Kaposi’s sarcoma (KS), primary effusion lymphoma (PEL), and multicentric Castleman’s disease (MCD) (Chang et al., 1994; Cesarman et al., 1995; Soulier et al., 1995). Like all herpesviruses, KSHV has two phases of the lifecycle, latent infection and lytic replication (Zhao et al., 2015; Liu et al., 2017). During KSHV latent infection, most of the viral genome is suppressed through DNA methylation, repressive histone modifications and other regulatory mechanisms which negatively regulate gene expression (Pantry and Medveczky, 2009; Gunther and Grundhoff, 2010; Lu et al., 2010; Toth et al., 2010; Purushothaman et al., 2015). Changes in the host cell microenvironment could reactivate the virus from latency to the lytic cycle, wherein the inhibitory epigenetic marks are replaced by active ones to allow for transcription of viral lytic genes (Ye, 2017; Uppal et al., 2018). Three recent studies revealed that the KSHV life cycle was affected by RNA N^6^-adenosine methylation epigenetic modification (Ye et al., 2017; Hesser et al., 2018; Tan et al., 2018). In the first report, the authors found that most KSHV transcripts undergo m^6^A modifications by experiments of MeRIP combined with qRT-PCR (Ye et al., 2017). Knockdown (KD) or functional inhibition of FTO using a reagent, named meclofenamic acid (MA), could enhance the expression of lytic gene ORF50 and ORF57, while KD of METTL3 or the blocking of m^6^A could abolish the expression of the two lytic genes and virion production. The splicing of pre-mRNA of *ORF50*, a key KSHV lytic switch gene, was inhibited when m^6^A deposition was blocked. Further research suggested that m^6^A positively regulates *ORF50* (*RTA*) RNA splicing through binding of the YTH domain containing 1 (YTHDC1) to identified m^6^A sites in *RTA* pre-mRNA and the cooperation with serine/arginine-rich splicing factor 3 (SRSF3) and SRSF10. RTA itself could induce m^6^A decoration and enhance its own pre-mRNA splicing. In short, m^6^A marks in the KSHV genome promoted lytic replication. In the second report, the KSHV-positive renal carcinoma cell line iSLK.219 during lytic reactivation went through transcriptome-wide m^6^A-sequencing, and the results revealed that the m^6^A modification was present across most viral transcripts. Depletion of the m^6^A writer METTL3 and the reader YTHDF2 significantly impaired virion production in iSLK.219 and iSLK.BAC16 cells, suggesting that the m^6^A pathway functioned in a pro-viral manner. In contrast, ORF50 protein expression was increased upon depletion of METTL3 in KSHV infected B cells, reflecting the anti-viral impacts of m^6^A in the KSHV life cycle (Hesser et al., 2018). The second report emphasized the result that the m^6^A pathway might play different roles in promoting or inhibiting viral gene expression depending on the cell-type analyzed. The last report found that KSHV transcripts contain abundant and highly conserved m^6^A modifications among different cell types and infection systems. The m^6^A reader protein YTHDF2 inhibited KSHV lytic replication by facilitating the degradation of viral lytic transcripts which is consistent with the second report. The lytic replication period of KSHV induced dynamic reprogramming of the epitranscriptome, regulating relevant pathways that control lytic replication (Tan et al., 2018). This report provided insights into the mechanism of KSHV-induced disease by helping us understand the changes of viral and cellular m^6^A modifications during KSHV latent and lytic infection. The three articles together suggest that m^6^A modifications in KSHV might play a positive role through different mechanisms or play different roles owing to different cell types during lytic replication.

As a member of the polyomavirus family, the gene expression of simian virus 40 (SV40) is regulated in an early phase, encoding the viral regulatory proteins, and a late phase, encoding the viral structural proteins. In 1979, SV40 was reported to contain some m^6^A groups in transcripts in the “late” region of the virus (Canaani et al., 1979). However, the specific location of these m^6^A groups was not identified and their functional roles have remained unclear. Recently, a report demonstrated that the overexpression of YTHDF2 induced faster viral replication, and larger viral plaques in BSC40 cells infected with SV40, whereas mutational inactivation of the endogenous YTHDF2 gene, or the m^6^A writer METTL3, had the contrary effect, suggesting a positive influence for m^6^A modification in the regulation of the SV40 life cycle (Tsai et al., 2018). The authors also mapped the sites of m^6^A residues on SV40 transcripts and identified two m^6^A sites on the viral early transcripts and eleven m^6^A sites within the SV40 late region. The authors observed that the mutant virus replicated more slowly than wild type SV40 when they inactivated most of the m^6^A addition sites on the SV40 late mRNAs using synonymous mutations. Together, these results suggest that the addition of m^6^A residues to the late SV40 transcripts played a positive role in viral gene expression and replication.

Hepatitis B virus infection is the leading cause of chronic hepatitis (Fu et al., 2016) and plays a role in the development of cirrhosis (Zhao et al., 2011) and hepatocellular carcinoma (Chen J. et al., 2009; Chen L. et al., 2009; Levrero et al., 2009). Imam et al. (2018) reported that HBV transcripts detected from both liver tissues of chronic HBV patients and HBV-expressing cells contain m^6^A. They observed that m^6^A located in HBV 3′UTRs reduced the stability of these RNAs, ultimately affecting the expression of their corresponding proteins. YTHDF proteins were found to bind HBV transcripts, and the depletion of them increased HBV protein expression, likely by changing the stability of the HBV transcript. Similar effects were observed when the m^6^A site within the 3′ epsilon loop of all HBV transcripts was inactivated. However, the authors found that m^6^A at the 5′epsilon loop, which is present only in pregenomic RNA (pgRNA), positively regulates pgRNA reverse transcription. Therefore, m^6^A regulates HBV RNAs in more than one way, hinging on its position in the RNA. The roles of m^6^A in different viral infections are summarized in Table 3.

**Table 3:** Roles of m^6^A in different viral infections.

Virus type	Roles of m^6^A in the viral infection	Mechanisms	Reference
IAV	The addition of m^6^A residues in IAV transcripts could enhance viral gene expression and revealed increased pathogenicity in mice.	m^6^A increased IAV replication and infectious particle production.	Courtney et al., 2017
HCV	m^6^A negatively regulates HCV infection.	YTHDF proteins relocalized at viral assembly sites to retain HCV RNA with m^6^A, leading to reduced HCV particle production.	Gokhale et al., 2016
ZIKV	m^6^A methylome inhibit ZIKV infection.	YTHDF family proteins bound to m^6^A sites of ZIKV and decreased the viral titer.	Lichinchi et al., 2016b
VSV	m^6^A modifications play a negative role in the life cycle of VSV.	DDX46 recruited ALKBH5 via DDX46’s DEAD helicase domain to erase the m^6^A modification of three important antiviral transcripts MAVS, TRAF3, and TRAF6 and this caused decreased production of type I interferons.	Zheng et al., 2017
EV71	m^6^A modifications in EV71 RNA played a positive role in viral replication.	The genomic copy numbers of EV71 RNA were significantly decreased by silencing METTL3 gene and increased by FTO gene depletion. METTL3 interacted with viral RdRp 3D protein and induced enhanced sumoylation and ubiquitination of 3D, and further affected replication of EV71	Hao et al., 2019
KSHV	m^6^A modifications in KSHV might play different roles owing to different cell types during lytic replication.	m^6^A pathway promoted the production of KSHV virions in iSLK.219 and iSLK.BAC16 cells. In contrast, m^6^A suppressed ORF50 protein expression in KSHV infected B cells.	Hesser et al., 2018; Tan et al., 2018; Ye et al., 2017
SV40	m^6^A modifications play a positive influence in the regulation of the SV40 life cycle.	m^6^A induced faster viral replication, and larger viral plaques in BSC40 cells infected with SV40.	Tsai et al., 2018
HBV	m^6^A regulates HBV RNAs in more than one way, hinging on its position in the RNA.	m^6^A located in HBV 3′UTRs and 3′ epsilon loop reduced the stability of these RNAs. However, m^6^A at the 5′epsilon loop, positively regulates pgRNA reverse transcription.	Imam et al., 2018

## Discussion on the Inconsistent Reports

The addition of m^6^A in viral mRNAs has both pro-viral and antiviral functions in distinct viral life cycles (He et al., 2011). The m^6^A modification enhanced the translation of viral late transcripts in SV40 while decreasing infectious HCV particle production (Gokhale et al., 2016; Tsai et al., 2018). Different reports studying the m^6^A effects in the same virus indicated that there exist discrepancies in the distribution of m^6^A sites and roles of m^6^A in the viral life cycle such as the abovementioned studies focusing on HIV-1, KSHV and HBV. The reasons for the contradictory results remain unclear in this field. One reason is the suggestion that m^6^A machinery functions in a cell type specific manner to either promote or inhibit KSHV gene expression (Hesser et al., 2018). The use of different cell types to study the m^6^A modification is one of the reasons for the different results. Some other reasons might contribute to different results obtained. First, the m^6^A decoration itself is dynamic in controlling mRNA fate and its ability to impact virtually every stage of host gene expression (Gonzales-van Horn and Sarnow, 2017; Roundtree et al., 2017a). Therefore, different experimental results are likely to result from different time points of sampling or different viral infection times. Second, the intracellular localizations of m^6^A modified by viral RNAs might influence the availability of host proteins, which might further result in different functions of m^6^A in different viruses. Third, the distinct m^6^A distribution sites of HIV-1 might be attributed to the sequencing-based methodologies used by each report which had different resolutions. For example, neither the m^6^A-seq nor the PA-m^6^A-seq can discriminate between m^6^A and m^6^Am (Tan and Gao, 2018), therefore leading to comprehensive distribution of the two types of modification when using the abovementioned m^6^A transcriptome-wide profiling techniques. Finally, the binding sites of the three m^6^A reader proteins YTHDF1, YTHDF2, and YTHDF3 identified by CLIP-seq analysis were different in the reports about HIV-1 (Ao et al., 2016; Riquelme-Barrios et al., 2018). Certain binding sites for YTHDF1, 2, and 3 do not line up with predicted m^6^A sites (Riquelme-Barrios et al., 2018), suggesting that cytoplasmic readers would also bind the HIV-1 gRNAs in an m^6^A-independent manner or that the viral transcripts possess additional m^6^A sites that have not yet been mapped. In general, the different approaches, detection strategies, cell lines and other reagents used in m^6^A-related studies might contribute to the distinct results obtained, which deserve to be clarified in the future.

## Future Research: m^6^A as a Potential Target for Antiviral Therapy

Given that the presence of m^6^A participates in the life cycles of multiple viruses, drugs that target this pathway could have the potential to be used to fight a range of viral illnesses. A drug, 3-deazaadenosine (DAA), which inhibits m^6^A addition through depleting intracellular levels of the methyl donor SAM (Bader et al., 1978; Fustin et al., 2013), is a potent inhibitor of IAV replication at doses that do not exert any apparent cytotoxic effects (Fischer et al., 1990). DAA has been reported to suppress a wide variety of viruses, not only *in vitro* culture but also *in vivo* in mice and in rats (Bader et al., 1978; Wyde et al., 1990; Bray et al., 2000; Kennedy et al., 2016; Courtney et al., 2017). In addition, a reagent, known as meclofenamic acid (MA), which can inhibit FTO demethylation by competition on m^6^A-containing substrate binding, has been demonstrated to enhance the expression of KSHV lytic genes (Ye et al., 2017). It is not difficult to conclude that developing new small molecule inhibitors or drugs targeting m^6^A “writers” or “erasers” might prove to be of great significance for antiviral therapies.

## Author Contributions

All authors listed have made a substantial, direct and intellectual contribution to the work, and approved it for publication. WD analyzed the literatures and studies and wrote the manuscript. JL designed this review and revised the manuscript. The rest of the authors assisted with the process of writing the manuscript.

## Conflict of Interest Statement

The authors declare that the research was conducted in the absence of any commercial or financial relationships that could be construed as a potential conflict of interest.

## Abbreviations

ALKBH5: AlkB Homolog 5, RNA Demethylase
CAPRIN1: cytoplasmic activation- and proliferation-associated protein 1
CLIP-seq: crosslinking-immunprecipitation and high-throughput sequencing
DENV: Dengue virus
eIF3: Eukaryotic Translation Initiation Factor 3
FMRP: Fragile X Mental Retardation Protein 1
FTO: fat mass and obesity-associated protein
G3BP1/2: GTPase Activating Protein (SH3 Domain) Binding Protein 1
HCV: hepatitis C virus
hnRNPA2/B1: heterogeneous nuclear ribonucleoprotein A2/B1
IGF2BP1: Insulin Like Growth Factor 2 MRNA Binding Protein 1
KIAA1429: Vir Like m^6^A methyltransferase associated protein
LC-MS: liquid chromatography-mass spectrometry
m^6^A: N6-methyladenosine
m^6^A-LAIC-seq: m^6^A level and isoform characterization sequencing
m^6^Am: 2-*O*-dimethyladenosine
m^6^A-seq: N6-methyladenosine-sequencing
MAVS: mitochondrial antiviral signaling protein
MeRIP-seq: methylated RNA immunoprecipitation sequening
METTL14: Methyltransferase Like 14
METTL16: Methyltransferase Like 16
METTL3: Methyltransferase Like 3
miCLIP: m^6^A individual nucleotide resolution cross-linking and immunoprecipitation
NXF1: nuclear RNA export factor 1
PA-m^6^A-seq: photo-cross-linking-assisted m^6^A sequencing
PAR-CLIP: photoactivatable ribonucleoside-enhanced crosslinking and immunoprecipitation
RBM15: RNA Binding Motif Protein 15
RBM42: RNA Binding Motif Protein 42
RSV: Rous sarcoma virus
SAM: *S*-adenosylmethionine
SRSF10: Serine and Arginine Rich Splicing Factor 10
SRSF3, Splicing Factor: Arginine/Serine-Rich 3
TRAF3: TNF Receptor Associated Factor 3
TRAF6: TNF Receptor Associated Factor 6
USP10: Ubiquitin-Specific-Processing Protease 10
VSV: vesicular stomatitis virus
WNV: West Nile virus
WTAP: WT1 associated protein
YFV: yellow fever virus
YTHDC1: YTH Domain-Containing Protein 1
YTHDC2: YTH Domain-Containing Protein 2
YTHDF1: YTH N(6)-Methyladenosine RNA Binding Protein 1
YTHDF2: YTH N(6)-Methyladenosine RNA Binding Protein 2
YTHDF3: YTH N(6)-Methyladenosine RNA Binding Protein 3
ZC3H13: Zinc Finger CCCH Domain-Containing Protein 13
ZIKV: Zika virus.
